# Steady-state approximations for Hodgkin-Huxley cell models: Reduction of order for uterine smooth muscle cell model

**DOI:** 10.1371/journal.pcbi.1011359

**Published:** 2023-08-30

**Authors:** Shawn A. Means, Mathias W. Roesler, Amy S. Garrett, Leo Cheng, Alys R. Clark

**Affiliations:** Auckland Bioengineering Institute, University of Auckland, Auckland, New Zealand; University of California San Diego, UNITED STATES

## Abstract

Multi-scale mathematical bioelectrical models of organs such as the uterus, stomach or heart present challenges both for accuracy and computational tractability. These multi-scale models are typically founded on models of biological cells derived from the classic Hodkgin-Huxley (HH) formalism. Ion channel behaviour is tracked with dynamical variables representing activation or inactivation of currents that relax to steady-state dependencies on cellular membrane voltage. Timescales for relaxation may be orders of magnitude faster than companion ion channel variables or phenomena of physiological interest for the entire cell (such as bursting sequences of action potentials) or the entire organ (such as electromechanical coordination). Exploiting these time scales with steady-state approximations for relatively fast-acting systems is a well-known but often overlooked approach as evidenced by recent published models. We thus investigate feasibility of an extensive reduction of order for an HH-type cell model with steady-state approximations to the full dynamical activation and inactivation ion channel variables. Our effort utilises a published comprehensive uterine smooth muscle cell model that encompasses 19 ordinary differential equations and 105 formulations overall. The numerous ion channel submodels in the published model exhibit relaxation times ranging from order 10^−1^ to 10^5^ milliseconds. Substitution of the faster dynamic variables with steady-state formulations demonstrates both an accurate reproduction of the full model and substantial improvements in time-to-solve, for test cases performed. Our demonstration here of an effective and relatively straightforward reduction method underlines the particular importance of considering time scales for model simplification before embarking on large-scale computations or parameter sweeps. As a preliminary complement to more intensive reduction of order methods such as parameter sensitivity and bifurcation analysis, this approach can rapidly and accurately improve computational tractability for challenging multi-scale organ modelling efforts.

## Author summary

Mathematical modeling of physiological organ systems encompassing intracellular to organ-wide behaviour grapple with intrinsically multi-scaled systems in both space and time. Significant computational challenges arise with their numerical solution, often substantially constraining feasibility of *in silico* investigations. Naturally, streamlining and reducing the mathematical complexity of these models while maintaining accurate reproduction of experimental data is a persistent concern. We present a straightforward method for exploiting multiple time scales reducing the order of models by way of steady-state approximations at the cellular level. Applied to a uterine smooth muscle cell model, we obtain substantial improvements in time to solve by around a factor of two while accurately reproducing full model results. Our successful demonstration here highlights the importance and relative ease of the method—that is not altogether unknown but often overlooked—with potential application across a wide variety of multi-scale organ modeling efforts.

## Introduction

Multi-scale mathematical models simulating electrical activity of muscular organs, such as the cardiac [1], gastro-intestinal [2] and uterine [3] systems, rely heavily on accurate descriptions of cellular functions. Striking a balance between realistic representations at the cell level and computational feasibility at the organ scale is a critical consideration in multi-scale modelling. Most biophysical cell models represent complex arrays of ion currents and transporters for comprehensive descriptions of their cellular functions [4]. However, scaling to the organ level often compels simplification of cell level models, with a challenge of retaining key functional contributors in a model without excessive complexity and computational intractability.

A classical example of model simplification is the FitzHugh-Nagumo (FN) reduced order version of the detailed Hodgkin-Huxley (HH) giant squid axon action potential model [5, 6]. The HH model exhibits multiple and wide-ranging time scales and FitzHugh exploited these time scale differences for the FN reduction. Other formal reduction of order methods such as sensitivity and bifurcation analysis are powerful and effective techniques, but often involve considerable effort. Typically encompassing high dimensional parameter spaces, local or global sensitivity analysis methods such as the Sobol or partial rank correlation coefficient [7] combined with dynamic reduction such as that presented by Parthimos, et al. [8] are often non-trivial.

Several multi-scale mathematical studies investigated organ-wide uterine function built on individual uterine smooth muscle cell (uSMC) modeling efforts—as recently reviewed by our group [9]. These cell models range from detailed and complex arrays of numerous ion currents and transporters [10–12] to minimal methods using the classic FN model [13]. Recent trends are toward comprehensive cell level models, but this can significantly complicate assembly and numerical solution when extended to full multi-scale organ models. Particularly, investigations of longer time scale phenomena such as uterine contractions during labour as compared to rapid time scales of cellular behaviour suggest the potential for simplification.

Ion channel submodels in uSMC models exhibit relaxation time scales ranging from less than ten milliseconds to hundreds of seconds, yet organ scale behaviour stretches out to orders of seconds and longer. In this study, we follow an approach inspired by that of FitzHugh [5] for simplifying a complex uSMC model into a more readily solvable system. Full dynamic variables with relatively fast relaxation times are exploited and replaced with steady-state approximations, greatly mitigating computational expense. This method is demonstrated on a comprehensive uSMC model published by Tong et al. [12]. Given the prevalence of HH-type cell models, however, this approach may be applied to a wide range of cell types such as gastro-intestinal [14], cardiac [15], or of course neuronal [5]. Recent multi-scale models for these systems could moreover benefit from consideration of time-scales for these easily overlooked steady-state approximations and corresponding improvements to computational expense [1, 2].

## Methods

### Reduction of order methodology

**Fig 1:**
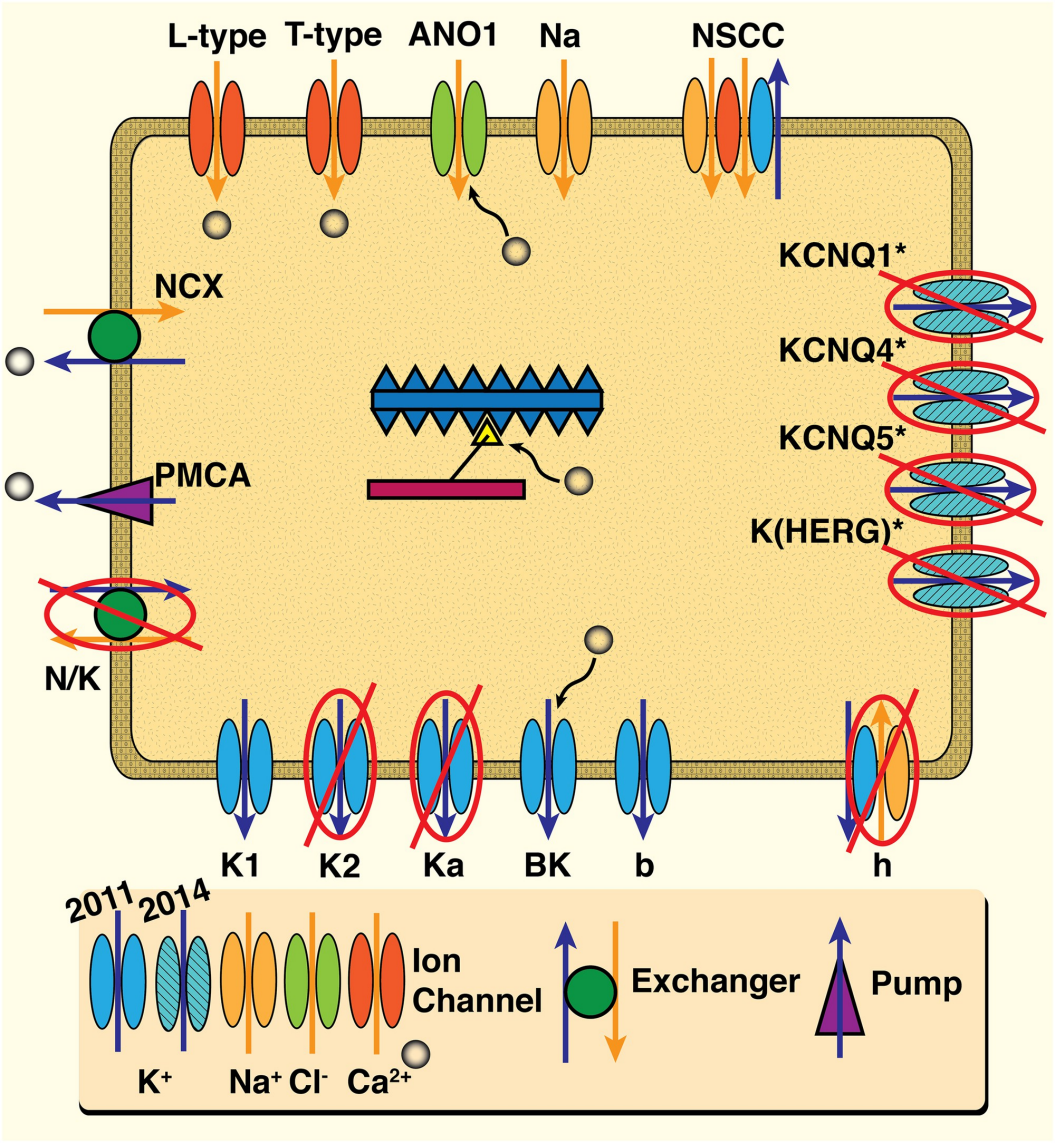
Schematic of Full Tong uSMC Model (FTM). All ion channel, exchanger and pump mechanisms of the 2011 model are presented, with the later added mechanisms of the 2014 version designated as shown. Note, only Ca^2+^ion concentrations dynamically vary in the Tong models; Na^+^, K^+^ and Cl^−^ are held fixed throughout. Ca^2+^concentrations modulate channel behaviour for the Cl^−^ (ANO1) and the K^+^ (BK) as well as the contractile force generating submodel illustrated in the centre. Mechanisms excluded from our final Reduced Tong Model (RTM) are noted with red crossouts.

We describe here our approach to simplifying a HH-type ion channel cell model based on the extensive effort of Tong, et al., both the initial 2011 and later updated 2014 versions [12, 16]. The complete published version of Tong, et al., 2011 we denote as the ‘Full Tong Model’ (FTM), and our reduced version as simply the ‘Reduced Tong Model’ (RTM). The schematic in Fig 1 shows all components of the FTM model, and the mechanisms that are ultimately removed in our analysis for the RTM. Avoiding exhaustive sensitivity and bifurcation analyses, the approach outlined here can inform other multi-scale organ modeling efforts, particularly for ion channel models exhibiting multiple time scales. The procedure utilised is two-fold: (*i*) eliminate channels in the cell model based on conductance. This occurs where FTM mechanisms are easily substituted by other mechanisms with modest modifications while maintaining qualitative reproduction of the full model; and (*ii*) timescale based reduction, in which we examine and replace the ion channel submodel dynamic variables remaining after the reduction of (*i*) exhibiting relatively fast time scales with their corresponding steady-state approximations. Note, we do not provide all formulations and expressions out of the full suite of 105 equations of the FTM; see Tong, et al. 2011 for details [12]. As the original FTM performed qualitative reproductions of experimental uSMC data; we do not overall apply quantitative metrics for comparing each output from our reduction effort. However, we do present both model results with relative differences upon completion of building the complete RTM.

Step (*i*) hinges on the observation that conductance of some channels in the FTM are substantially larger than others; see Table 1. Experimentation with a select reduced suite of channel submodels and variations of conductances suggested elimination of several K^+^ channels in the FTM; we detail this below in the section on Elimination of Channels based on Conductance.

**Table 1:** Conductances for the FTM. Items as taken from published values given in Tong, et al., 2011 including channels in the Tong, et al. 2014 version [16]. Ranges provided where suitable or used in original publication(s). Values in the CellML implementation of the model for reproduction of Tong, et al.’s Fig 12 are also shown, as well as equation numbers for the 2011 model (see the physiome repository). Final column indicates inclusion in the RTM; see text for further details.

Conductance Item	Symbol	Tong: Range (nS/pF)	CellML: Fig 12	Eqn. #	RTM Inclusion
Na^+^Channel	${\bar{g}}_{Na}$	0.028–0.125	0.0 (inactive)	20-27	✓
Ca^2+^Channel L-type	${\bar{g}}_{CaL}$	0.6		10-19	✓
Ca^2+^Channel T-type	${\bar{g}}_{CaT}$	0.058		28-34	✓
K^+^ Channels All	${\bar{g}}_{K}$	0.8			
K^+^ Channel ‘1’	${\bar{g}}_{K1}$	0.65	0.52	40-49	✓
K^+^ Channel ‘2;	${\bar{g}}_{K2}$	0.04	0.032	50-58	
K^+^ Channel ‘a’	${\bar{g}}_{Ka}$	0.2	0.16	59-65	
K^+^ Channel ‘BK’	${\bar{g}}_{BK}$	0.3${\bar{g}}_{K}$		66-78	✓
K^+^ Channel ‘b’	${\bar{g}}_{b}$	0.004		79	
K^+^ Channel ‘h’	${\bar{g}}_{h}$	0.0542		35-39	
Cl^−^ Channel An01	${\bar{g}}_{Cl}$	0.1875		80-86	✓
Na^+^, Ca^2+^, K^+^ Channel NSCC	${\bar{g}}_{NS}$	0.0123		87-92	✓
Na^+^K^+^ Exchanger	${\bar{g}}_{NaK}$	1.7	0.0403 (max)	93-96	
Na^+^Ca^2+^Exchanger	calculated	n/a	-0.0587 (min)	99-103	✓
K^+^ Channel KNCQ1 (2014)	${\bar{g}}_{KCNQ1}$	0–0.036	n/a		
K^+^ Channel KNCQ4 (2014)	${\bar{g}}_{KCNQ4}$	0–0.04272	n/a		
K^+^ Channel KNCQ5 (2014)	${\bar{g}}_{KCNQ5}$	0–0.0208	n/a		
K^+^ Channel KhERG (2014)	${\bar{g}}_{KhERG}$	0–0.176	n/a		

**Fig 2:**
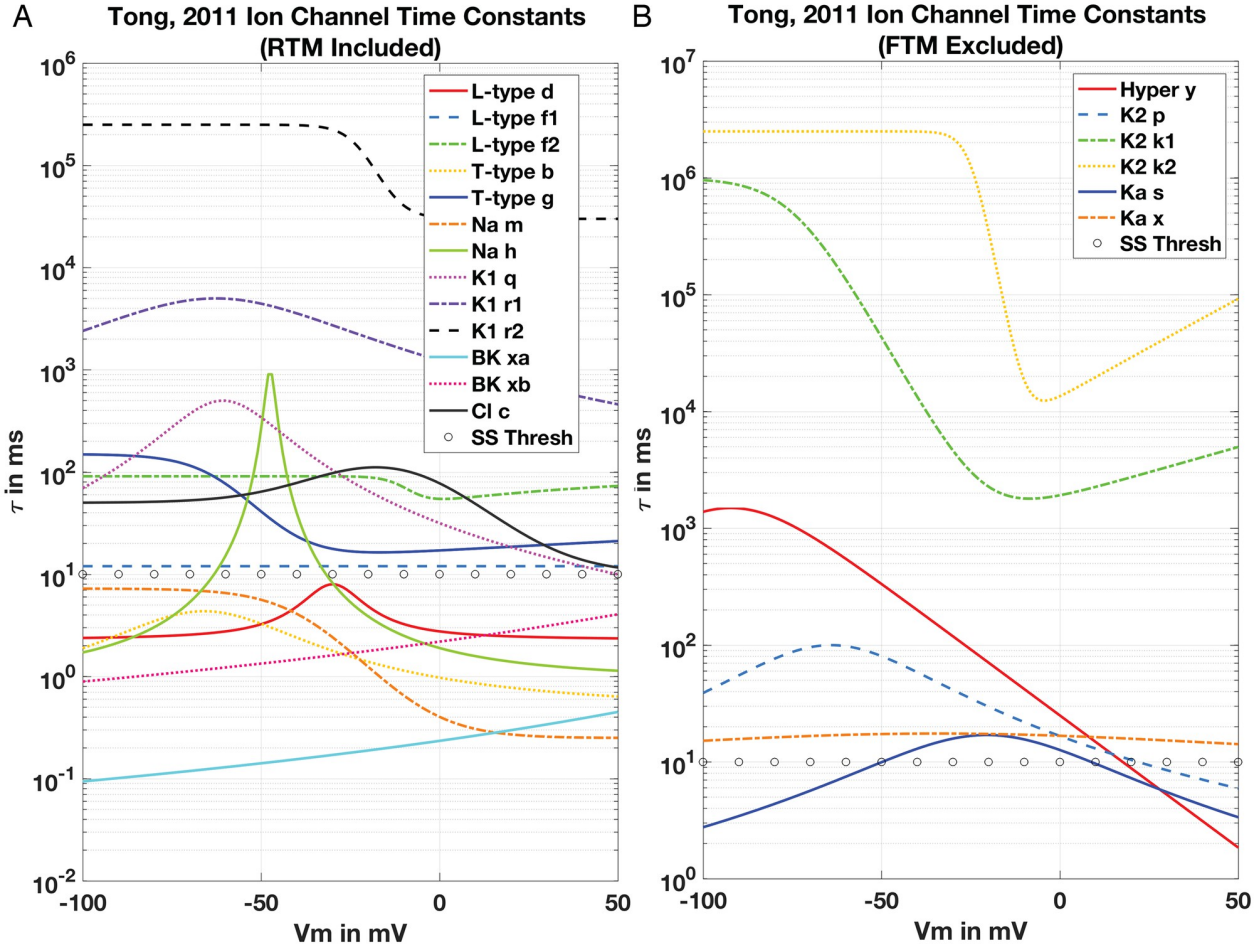
Relaxation time scales ion channel submodels. Panel (A). Suite of *τ* values as given for the RTM family of ion channels and the corresponding activation and inactivation variables. Note extraordinary range on logarithmic scale over six orders of magnitude from 10^−1^ milliseconds for the *BK*-channel *xb* variable (teal trace) to as high as 100 seconds for the K^+^ channel variable *r*_1_ (dashed black trace). Each variation over *V*_*m*_ are computed from the functional dependencies as given in the FTM, and selected channels in the RTM version appear here as described in text. The rough threshold for consideration of steady-state approximation partitioning the suite of these variables is at 10 *ms* (open circles), that is also sensitive to the range of *V*_*m*_ encountered during simulation. Panel (B). Corresponding suite of *τ* values for the FTM but excluded mechanisms from the final RTM. Note extraordinary range of time scales but shifted up around two orders of magnitude compared with the RTM suite.

Step (*ii*) is inspired by the observed range of time scales. Ion channel time constants associated with the FTM span six orders of magnitude, from 10^−1^ milliseconds (the Ca^2+^ -modulated *BK*-channel) to 100 seconds (the *K*_1_ channel), illustrated in Fig 2A. This suggests, that inspired by the FN reduction of HH [5] there is scope to replace dynamical variables—Ordinary Differential Equations (ODEs)—in the FTM with their steady-state formulations. This aspect hinges on the form of channel models we inherit from the seminal work of HH, whereby the ion current is computed thus for a generic ion ‘*x*’:

$$\begin{array}{cc} I_{x} & =g_{x}m_{x}h_{x}\left(V_{m}-E_{x}\right),\\ E_{x} & =\frac{RT}{nF}\;\text{ln}\;[\frac{X_{o}}{X_{i}}], \end{array}$$ (1)

for a conductance of *g*_*x*_, where activation and inactivation variables are *m*_*x*_ and *h*_*x*_, respectively. Driving force is given by the difference of membrane voltage (*V*_*m*_) from the, typically, Nernst equilibrium for ion ‘*x*’ (*E*_*x*_), and valence *n* along with the gas constant *R*, temperature *T* and Faradays constant, *F*. The dynamic ODE variables *m*_*x*_ and *h*_*x*_ determining the current are classically described as:

$$\frac{dm}{dt}=\frac{m_{\infty }-m}{\tau_{m}};m_{\infty },\tau_{m}\propto V_{m},$$ (2)

the activation variable, *m*_*x*_, and similarly for *h*_*x*_. These variables relax at some time scale, *τ*_*m*_, to the steady-state value *m*_∞_, for instance. Often the steady-state and relaxation times are dependent on membrane voltage, *V*_*m*_, as with the Na^+^ channel and possibly ions or ligands such as Ca^2+^ in the Ca^2+^-modulated *BK* K^+^ channel.

Typically, numerical simulations over time scales of seconds or longer set time step sizes to around *dt* = 0.01 seconds or 10 milliseconds—depending on the dynamics of the system of interest—and can rise given enough numerical stability. However, organ-wide uterine functions are dramatically longer [9]. Therefore we considered substituting the dynamic variables (*i.e*., *m*_*x*_) relaxing at or below a threshold (*i.e*., *τ*_*m*_ ≤ *dt* = 0.01 s) with their corresponding steady-state values (*i.e*., *m*_∞_(*V*_*m*_)). We examined these substitutions in turn for multiple ion channel submodels as noted below in section Steady State Approximation.

### Summary of the Tong uSMC model

The complete FTM encompasses a total of nineteen ODEs, describing activation and inactivation variables for nine ion channels. Of these channels, the FTM includes two Ca^2+^ (L-type *CaL*, T-type *CaT*), one voltage-gated Na^+^, one Cl^−^ (the *ANO*1) and five K^+^ channels (*K*_1_, *K*_2_, *K*_*a*_, *BK* and *b*, or background current) with two additional channels permeable to either both Na^+^ and K^+^ (the ‘*h*’ hyperpolarisation) or Ca^2+^ as well (the *NSCC* with no ODEs). Four additional K^+^ channels appears in the 2014 Tong, et al. model [16]. Membrane voltage, *V*_*m*_, is then computed by

$$\begin{array}{cc} \frac{dV_{m}}{dt} & =-\frac{1}{C_{m}}[\sum (I_{ion}+I_{exchanger})]+I_{app}\\ I_{ion} & =I_{CaL}+I_{CaT}+I_{Na}+I_{K}+I_{h}+I_{NSCC}\\ I_{K} & =I_{K1}+I_{K2}+I_{Ka}+I_{BK}+I_{b}\\ I_{exchanger} & =I_{NCX}+I_{N/K}, \end{array}$$ (3)

where *C*_*m*_ is membrane capacitance, *I*_*ion*_ is the current through all ion channels, *I*_*exchanger*_ is the current through exchangers (the Na^+^-Ca^2+^ exchanger, *NCX*, and the Na^+^-K^+^ exchanger, *N*/*K*), and *I*_*app*_ is the applied stimulus. Current through each of the channels/exchangers in Table 1 is represented by *I*_*x*_, where *x* is that channel’s identifier. Note the FTM does not dynamically track Na^+^ or K^+^ concentrations; the only influence of K^+^ and Na^+^channels is by way of currents in the expression for *V*_*m*_. Alternatively, [Ca^2+^]_*i*_ is tracked dynamically through action of channel influx and pump or exchanger efflux thus:

$$\frac{dCi}{dt}=\beta (J_{CaL}+J_{CaT}+J_{NSCC})-J_{NCX}-J_{PMCA}.$$ (4)

where extruder action such as the plasma-membrane Ca^2+^ ATP-ase (*PMCA*) and the *NCX* remove cytosolic Ca^2+^. Free cytosolic [Ca^2+^]_*i*_ is a small fraction of actual Ca^2+^ influx due to wide arrays of intracellular Ca^2+^ buffering mechanisms such as the mobile protein calmodulin or uptake via the Sarco/Endoplasmic Reticulum ATP-ase (SERCA) into organelle Ca^2+^ reservoirs. *β* represents this effect with a simple constant fraction and is set to 0.015, which they reduced from the cited source of Standen & Stanfield, 1982 [17]. Presumably, Tong, et al. lowered the Standen & Stanfield neuronal *β* for improved fits to uSMC data; we also consider *β* a free parameter for Ca^2+^ fitting purposes.

### Standard Simulation Protocol (SSP)

We compare our RTM to the FTM by using what we define as the standard simulation protocol (SSP). We target the stimulated behaviours shown in Fig 12A of Tong et al. [12], which aimed to replicate experimental observations reported in Fig 2A of Okabe, et al. [18]. This SSP is an applied current of *I*_*app*_ = −0.5 pA/pF over 2 seconds and simulations of model responses out to a *t*_*max*_ of 10 seconds. All tests shown are with this strength and duration *I*_*app*_ as well as *t*_*max*_ except where otherwise stated. The FTM equations and parameters were provided by a CellML implementation of the Tong, et al. 2011 published model in the Physiome Model Repository (PMR [19]). The PMR provides curated versions of published cell models that reproduce results presented in the original publication. Table 1 lists conductances presented in both the original Tong, et al. 2011 publication and the CellML values utilised for reproduction of Fig 12A in Tong, et al. We utilised two numerical platforms: OpenCOR for importing the CellML model descriptions and full model simulations and comparisons between the FTM and RTM, and MATLAB for generation of *I-V* curves of the individual ion channel submodels. Numerical solutions of the FTM and RTM system of ODEs performed in OpenCOR [20] utilised CVODE with the backwards differentiation formula (BDF) time integrator and tolerances (absolute and relative) set to 1*e*^−7^ and a maximum adaptive *dt* set to 0.1 ms. MATLAB assembly of *I-V* curves utilised ode23s and both tolerances set to 1*e*^−10^, with maximum *T* as noted. All single-cell simulations presented were performed on a macbook pro laptop (2.8 GHz Intel i7 with 4 cores and hyperthreading).

Pilot simulations for 2D & 3D tissue geometries were performed on a 144 CPU compute workstation (3.1 GHz Intel Xeon 6254) using a single-thread and the Chaste [21] implementation of the CVODE solver with default options and adaptive time stepping. We utilised a simple 2D ‘tissue’ (1 mm square; 121 nodes with 200 quadrilateral elements) and a hollow 3D cylindrical tube approximating a rat uterine horn (length 10 mm and 0.3 mm thick with 1910 nodes in a 4074 tetrahedral elements). The SSP stimulus of -0.5 pA/pF was applied in a sub-region: 0.2 x 0.01 mm (2D) and 1.0 mm along the tubular axis (3D). Electrical signal diffusion was set at a rate of 0.013 mS/cm (2D) and 250.0 mS/cm (3D).

## Model reduction

### Elimination of channels based on conductance

Table 1 shows the K^+^ channels in the FTM. One variant (termed K^+^ Channel ‘1’, or *K*_1_) clearly dominates the overall current: with ${\bar{g}}_{K1}=0.65$ nS/pF. The K^+^ Channel ‘a’ (*K*_*a*_) is a distant second with ${\bar{g}}_{Ka}=0.2$ nS/pF. Tong noted the *K*_1_ as the most influential of all the K^+^ channels and Knock, et al. observed the *K*_1_ exhibits 67% of total K^+^ current in the myometrium [22], while the *K*_2_ at around 23%, and the Ca^2+^-triggered *BK* rounding out the remaining 10%. Cells were observed to deploy either the *K*_1_ or the *K*_2_, but overall the *K*_1_ emerged as the more typically expressed in uSMC [22]. Atia et al. also predicted a significant role in uSMC for a functionally similar channel to the *K*1, known as the *Kv*2.1 [23], reinforcing the report of Knock and the model of Tong. We thus explore the potential for the *K*_1_ (or *Kv*2.1) as a representative K^+^ channel for the entire K^+^ current, or *I*_*K*_ of Eq (3), in the FTM for our reduction effort. Note, we do not alter the dynamics of the *K*_1_ channel model given to us by Tong et al.—only the conductance values are varied.

#### Eliminating K^+^ channel ‘a’

**Fig 3:**
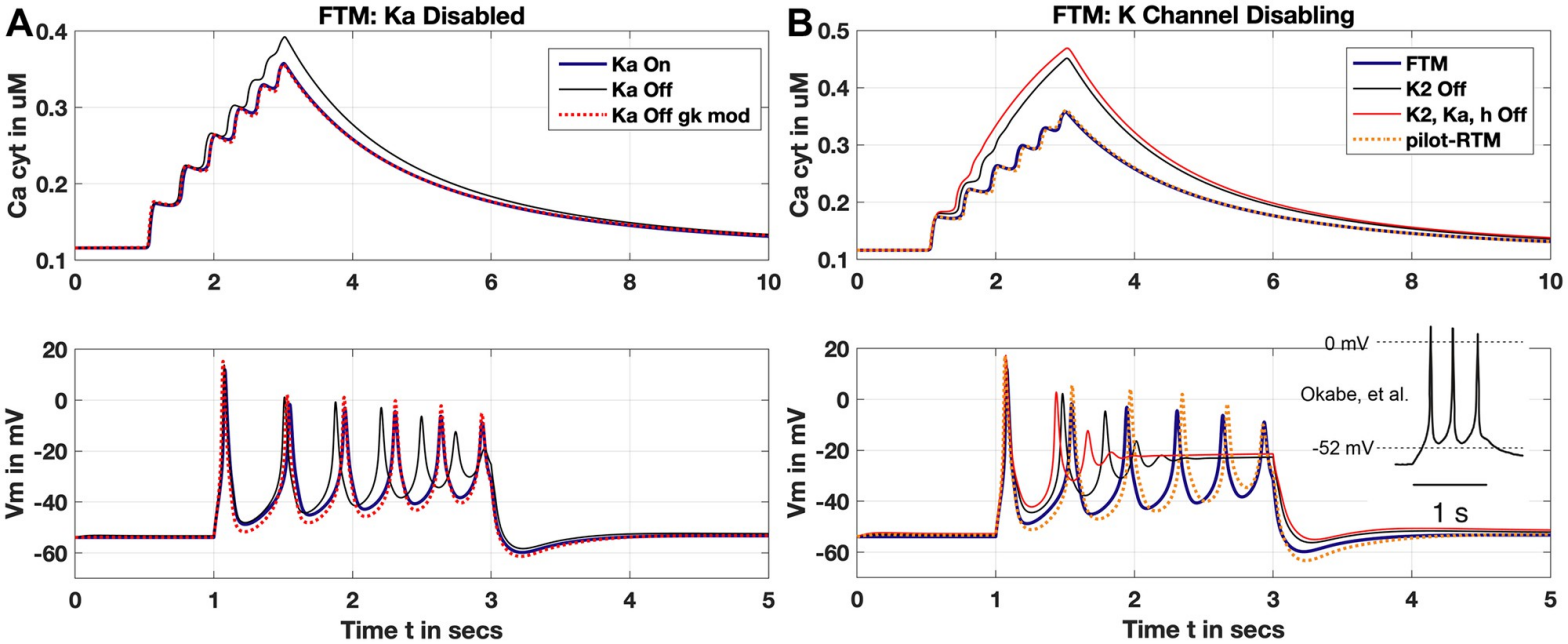
K^+^ current inactivation. (Panel A) Disabling *K*_*a*_ current. FTM result with identical test protocol to the SSP reproduced by the CellML implementation (blue trace). Both [Ca^2+^]_*i*_ and *V*_*m*_ (upper and lower Panes, respectively; note varying time scales) display similarity to FTM with *K*_*a*_ disabled and no modifications otherwise (black trace). Altered conductances for *K*_1_ and *I*_*Na*_ of 0.598 and 0.07 nS/pF, respectively, along with increased fraction of free (unbuffered) Ca^2+^ by 2.5% (*β* = 0.0154) presents improved qualitative fit to FTM (red trace, dotted). (Panel B) Disabling *K*_2_, *K*_*a*_ and *h* utilising the SSP as in Panel A. Disabling only *K*_2_ (black trace), exhibits significant rise in [Ca^2+^]_*i*_ (upper pane), and truncated APs settling into elevated plateau *V*_*m*_ (lower pane) compared to FTM result (blue trace). Disabling *K*_2_, *K*_*a*_ and *h* shows similar behaviour (red trace). Alterations to *K*_1_ and *I*_*Na*_ of 0.655 and 0.05125 nS/pF, respectively, with *β* = 0.0146 improve qualitative fit to FTM (pilot-RTM, orange dotted). Experimental trace adapted from Tong, 2011 Fig 12A originally via Okabe, et al. [18] (inset, Panel B) showing pilot-RTM reproduces observed *V*_*m*_ peaks above 0 mV over 1 second. ‘Pilot-RTM’ here refers to interim reduced model; see text for more.

We initially test disabling of the K^+^ current, *K*_*a*_ due to prominence of ${\bar{g}}_{Ka}$ over remaining K^+^ channels without *K*_1_ (see Table 1) and compare the simulated result of the FTM for the SSP (See Fig 3A). Deactivation of *K*_*a*_ presents a similar result to the FTM but with noticeable deviations. This includes an accelerated frequency of action potentials (APs), diminished *V*_*m*_ amplitude and higher peak [Ca^2+^]_*i*_. Modifying the conductances for *I*_*K*1_ and *I*_*Na*_ we obtain an improved fit, with slightly overshot peaks and valleys of amplitudes but excellent temporal alignment of APs. For *I*_*K*1_, we increased above the CellML value to *g*_*K*1_ = 0.598 nS/pF as well as raising *g*_*Na*_ to 0.07 nS/pF—still well within the range given in Table 1. Additionally, raising the constant fraction value for free unbuffered calcium, *β*, by a mere 2.5% tightly aligns the [Ca^2+^]_*i*_ trace during the test stimulus peaks and out to steady-state as shown.

#### Eliminating K^+^ channel ‘2’

Although the conductance of *K*_*a*_ is substantially higher than that for the *K*_2_ channel in the FTM (0.2 versus 0.04 nS/pF), the impact of disabling the *K*_2_ is noticeably more significant (Fig 3B). Without the *I*_*K*2_, under the SSP, APs are damped into a higher steady-state *V*_*m*_ and [Ca^2+^]_*i*_ rises considerably higher to around 450 nM. Inactivating *I*_*Ka*_ and *I*_*h*_ as well enhances these effects without substantial overall change; the *K*_2_ channel is thus more influential despite the order of magnitude less conductance than the *K*_*a*_. This is explained by the time scales for current activity of the *K*_2_ and the *K*_*a*_ channels as presented in the Tong, 2011 current data (see their Figures 6E and 7C [12]). The *K*_2_ remains active over the course of seconds whereas the *K*_*a*_ shuts down within 50-100 ms; hence the *K*_2_’s apparently greater impact we see here. Nevertheless, modest modifications to channel conductances and the *β* produce the qualitatively good alignments for this ‘pilot-RTM’ between AP peaks and the corresponding stair-step rise in [Ca^2+^]_*i*_ concentration. We only present the interim RTM here including only conductance variations without any steady-state modifications as done below; this we denote the ‘pilot-RTM’. Moreover, the pilot-RTM results shown exclude the *N*/*K* and *NCX* exchanger influence on *V*_*m*_ (see crossouts in Fig 1), although *J*_*NCX*_ still actively extrudes [Ca^2+^]_*i*_. Modest adjustments to the *I*_*K*1_ and *I*_*Na*_ thus compensate for and reproduce the overall result of the FTM without the full suite of K^+^ mechanisms.

The experimental trace of Okabe et al. is shown for comparison (Panel B, inset Fig 3), as utilised in Fig 12A of Tong, et al. Our pilot-RTM result qualitatively reproduces well the reported Okabe trace [18]. A similar spike train of APs and peak AP levels exceed 0 mV over the 1 second duration of their stimulus test with similar conditions, whereas the FTM *V*_*m*_ levels all fall under 0 mV after the initial AP. However, temporally the AP peaks are slightly delayed for the pilot-RTM from the Okabe trace; this alignment is improved below with steady-state approximations.

#### Other K^+^ mechanisms

Remaining K^+^ currents in the 2011 FTM such as the background, *I*_*b*_, and the Ca^2+^-modulated *BK* sit at opposite ends of the conductance range. *I*_*b*_ is smaller than all others by an order of magnitude and the *BK* is at 30% of total ${\bar{g}}_{K}$ (compare Table 1). Despite the marginal magnitude of *g*_*b*_, it maintains resting *V*_*m*_ at around -58 mV acting as a collective K^+^ channel representative [12]. Disabling *I*_*b*_ slowly depolarises *V*_*m*_ until the L-type channel is triggered and bursting occurs. Alternatively, the *NSCC* also maintains resting *V*_*m*_ but in the opposite direction, by preventing *V*_*m*_ from falling to the equilibrium potential for *I*_*b*_ at *E*_*K*_ = −84 mV. Both functional forms of *I*_*b*_ and *NSCC* are computationally straightforward, and we thus retain both mechanisms for resting *V*_*m*_ maintenance in the RTM.

The *BK*, however, is a rather different concern. Deactivation of *I*_*BK*_ produces similar results as inactivation of *I*_*K*2_, with truncated APs leading to a depolarised steady-state *V*_*m*_ during stimulus, and a significant rise in free [Ca^2+^]_*i*_ over the FTM under the SSP, that can be mitigated with adjustments to *g*_*K*1_. However, the *BK* channel dynamic variables as displayed in Fig 2A relax faster than any other mechanism in the FTM. Both the *τ*_*xa*_ and *τ*_*xb*_ scales are well under 10 ms over the entire range of -100 to 50 mV—beyond the physiological *V*_*m*_ for uSMC. This rapid relaxation appears ripe for exploitation and retention of the *BK* in our RTM. Particularly, of great interest to multi-scale modeling efforts are Ca^2+^ dynamics in the uSMC and Ca^2+^ modulation of the *BK*, the *ANO*1, contractile force generation, and myriad other elements subject to Ca^2+^ fluctuation. Assembly of an RTM that captures essential behaviour while reducing computational complexity by elimination of ODE variables in the model achieves this aim quite effectively, which we describe in more detail next with Steady-state Approximation.

### Steady-state approximation

Uterine organ-wide functions in clinical settings occur over seconds to hours and months [24] that vastly eclipse behaviour of individual channel subunits. Organ scale models typically attempt to reflect clinical measurements, and so the timescales of importance in these models are seconds to hours and beyond.

#### The K^+^ BK channel

The Ca^2+^-modulated K^+^ channel, or ‘big’ current *BK*, adapts rapidly to rising *V*_*m*_ with relaxation *τ* for the *BK* dynamic variables, *x*_*α*_ and *x*_*β*_, reaching at most about 4 ms (*V*_*m*_ ≤ 50 mV, see Fig 2A). These *τ* are further independent of [Ca^2+^]_*i*_ and the steady-state (SS) depictions of the *BK* current are dependent only on *V*_*m*_:

$$\begin{array}{cc} I_{BK} & ={\bar{g}}_{BK}\left(p_{\alpha }x_{\alpha }+p_{\beta }x_{\beta }\right)\times \left(V_{m}-E_{K}\right)\\ x_{i}^{ss} & =1/[1+\text{exp}(\frac{Fz_{i}\left(c_{i}\right)(V_{m}-V_{i}\left(c_{i}\right)}{RT})];\left(\text{dimless}\right);i\in \left[\alpha ,\beta \right]\\ \tau_{\alpha } & =2.41/(1+(\frac{V_{m}-158.78}{-52.15}{)}^{2})\left(\text{in}\;\text{ms}\right)\\ \tau_{\beta } & =13.8/(1+(\frac{V_{m}-153.02}{66.5}{)}^{2})\left(\text{in}\;\text{ms}\right), \end{array}$$ (5)

for fractional activation of the two variables *x*_*α*_ and *x*_*β*_ by the proportions *p*_*α*_ and *p*_*β*_, respectively, and a Ca^2+^ dependency, *z*_*i*_, not detailed here. Dynamic ODEs tracking these two activation variables, *x*_*α*_ and *x*_*β*_, are given by the standard formulation

$$\begin{array}{cc} \frac{dx_{i}}{dt} & =\frac{x_{i}^{ss}-x_{i}}{\tau_{i}}\\ \Rightarrow x_{i}\left(T\right) & =x_{i}^{ss}-\left(x_{i}^{ss}-x_{i0}\right)\;\text{exp}\;(\frac{-T}{\tau_{i}});i\in \left[\alpha ,\beta \right], \end{array}$$ (6)

for some time span *t* ∈ [0, *T*]. We omit numerous details such as with *z*_*i*_ computed in $x_{i}^{ss}$; see Tong, et al. for complete formulations. Clearly, the ratio of *T* and *τ*_*i*_ determine accuracy of approximating *x*_*i*_ with $x_{i}^{ss}$. Utilising $x_{i}^{ss}$ instead of the full ODE when simulating beyond about *T* = 10 ms should be adequate for seconds-scaled simulations given *dt* at around 0.01 seconds, and the extraordinarily rapid relaxation times of *τ*_*α*_ and *τ*_*β*_ under 10 ms.

**Fig 4:**
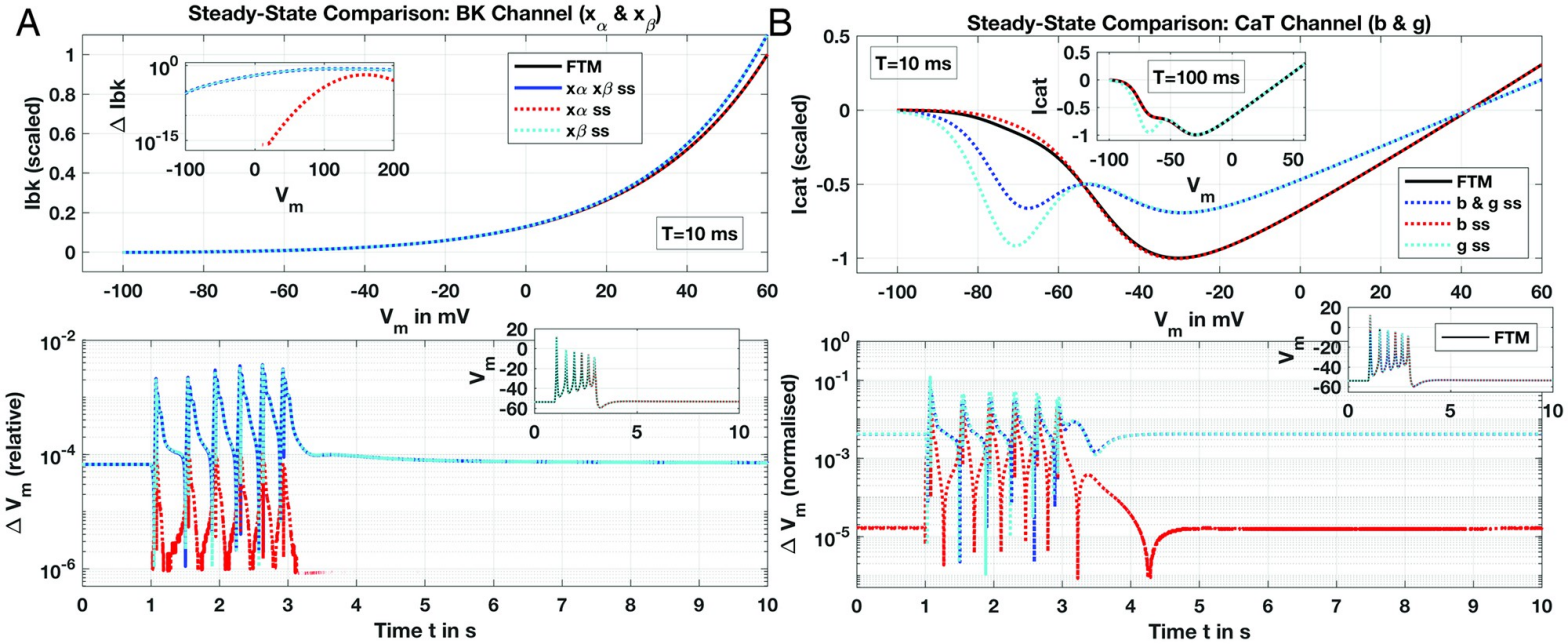
Steady-state approximation comparisons with full ODE solutions for *I* − *V* curves and *V*_*m*_ results from FTM simulations; *BK* (Panel A) and T-type (Panel B) channels. Full ODE solutions displayed with black traces (labeled ‘FTM’) and hybrid SS-ODE or full SS solutions with dotted and/or coloured traces as noted. For currents of channels, numerically-solved ODEs and SS approximation computations for each channel performed over a range of *V*_*m*_ for *t* ∈ [0, *T*] with *T* = 10 or 100 ms as noted, and corresponding currents, *I*_*x*_, shown, with conductances, ${\bar{g}}_{x}$ all set to 1 (Panels *A, B*, upper panes). Resulting *I*_*x*_ for each channel scaled by maximum current computed with ODE solutions: outward for *BK* and inward for T-type. SSP simulated *V*_*m*_ presented for both full ODE and SS variables of the FTM (Panels *a, b*, lower inset panes) with Δ*V*_*m*_ for each result as shown. (Panel a) *BK* channel variables *x*_*α*_, *x*_*β*_ computed either with ODE solver (black trace) or SS approximation for current (scaled *I*_*bk*_, upper pane) and relative difference in current (Δ*I*_*bk*_, upper pane inset) for comparison. Note legend traces apply to both panes: blue trace for both *x*_*α*_ and *x*_*β*_ approximated with SS, and red and cyan dotted for each SS-approximated variable in solo. Lower pane presents Δ*V*_*m*_ for SSP results (*V*_*m*_ shown in inset) with combinations of full ODE (FTM, black trace) and SS-approximations as annotated. (Panel b) *CaT* channel variables *b* and *g*. *I*_*cat*_ (scaled) in upper pane over scanned *V*_*m*_ range with *T* = 10 ms and *T* = 100 ms (inset) for full ODE (black trace, FTM) and SS-approximation for both *b* and *g* (blue dotted) or each variable SS-approximated in solo as noted (red, cyan). Legend traces apply to both panes. Lower pane: Δ*V*_*m*_ for SSP simulation with *CaT* channel either full ODE (FTM, black trace) or combinations of SS-approximations as noted; corresponding *V*_*m*_ results for SSP of each ODE-SS configuration displayed in inset. See SSP description for numerical details.

Numerical scans solving the ODE of Eq 6 over *V*_*m*_ ∈ [−100, 60] out to *T* = 10 ms display excellent agreement between full ODE solutions for both *x*_*α*_ and *x*_*β*_ and their corresponding SS approximations (see Fig 4A), computing current, *I*_*BK*_, with a normalised conductance, i.e., ${\bar{g}}_{BK}=1$ as given in Eq 5. Deviation for *I*_*BK*_ (Δ*I*_*BK*_) over the extended *V*_*m*_ range out to 200 mV (well above experimental observation) is at most about 0.2 (relative, normalised), and machine-*ϵ* errors for physiological *V*_*m*_ below about 20 mV. FTM results for the SSP utilising these $x_{i}^{ss}$ reproducing the result of Tong, Fig 12 are virtually identical to the full *BK* ODE model, with relative *ΔV*_*m*_ under 1% throughout for all combinations of SS approximations. The *τ* for both *BK* variables that are comfortably under 10 ms within physiological ranges of *V*_*m*_ ∈ [−80, 20] mV for uSMC as noted in Table 2 enable this result.

**Table 2:** RTM Channel subunit relaxation time scales per variable. Two ranges of *V*_*m*_ presented: beyond the physiological range of uSMC ([-100,200]) and within ([-80, 20]). Maximum values given correspond to plots shown in Fig 2.

Channel	Variable	Max(*τ*) in ms *V*_*m*_ ∈ [−100, 200] mV	Max(*τ*) in ms *V*_*m*_ ∈ [−80, 20] mV
K^+^ ‘BK’	*x*_*α*_, Activation	2.41	0.3
K^+^ ‘BK’	*x*_*β*_, Activation	13.80	2.76
Ca^2+^‘T-Type’	*b*, Activation	4.35	4.35
Ca^2+^‘T-Type’	*g*, Inactivation	148.89	137.88
Ca^2+^‘L-Type’	*d*, Activation	7.99	7.99
Ca^2+^‘L-Type’	*f*_1_, Inactivation	12.00	12.00
Ca^2+^‘L-Type’	*f*_2_, Inactivation	90.97	90.97
Na^+^	*m*, Activation	7.24	7.15
Na^+^	*h*, Inactivation	903.46	903.46
K^+^ K1	*q*, Activation		499.83
K^+^ K1	*r*_1_, Inactivation		4.9e3
K^+^ K1	*r*_2_, Inactivation		2.5e5
Cl^−^ ANO1	*c*, Activation		111.27

#### The T-Type Ca^2+^ channel

Relative to the L-type ${\bar{g}}_{CaL}$, T-type conductance ${\bar{g}}_{CaT}$ is substantially lower (see Table 1) manifesting currents at around two orders of magnitude less for *CaT* than the L-type during FTM SSP simulations. Doubling ${\bar{g}}_{CaT}$ still holds *I*_*CaT*_ at one-tenth of *I*_*CaL*_. Interestingly, setting the already low ${\bar{g}}_{CaT}$ to zero effectively eliminates two AP pulses from the SSP FTM simulation and reduces [Ca^2+^]_*i*_ considerably by ∼100 nM. The *CaT* maintains a trickling current reversing roles of significance at resting *V*_*m*_, with *I*_*CaT*_ ∼ −0.05 pA/pF and *I*_*CaL*_ ∼ −0.007 pA/pF. Thus, the *CaT* shapes behaviour at resting and stimulated *V*_*m*_, and we hence explore its steady-state simplification.

Analogously for the *CaT* channel with activation variable *b* and inactivation *g*, with their corresponding SS and *τ* dependencies on *V*_*m*_:

$$\begin{array}{cc} I_{CaT} & ={\bar{g}}_{CaT}b^{2}g\left(V_{m}-E_{CaT}\right)\\ b^{ss} & =1/(1+\text{exp}(\frac{-(V_{m}+54.23)}{9.88}))\left(\text{dimless}\right)\\ g^{ss} & =0.02+0.98/(1+\text{exp}(\frac{V_{m}+72.98}{4.64}))\left(\text{dimless}\right)\\ \tau_{b} & =0.45+3.9/(1+(\frac{V_{m}+66}{26}{)}^{2})\left(\text{in}\;\text{ms}\right)\\ \tau_{g} & =150-150/[(1+\text{exp}(\frac{V_{m}-417.43}{203.18}))(1+\text{exp}(\frac{-(V_{m}+61.11)}{8.07}))]\left(\text{in}\;\text{ms}\right), \end{array}$$ (7)

the *τ*_*b*_ remains below 10 ms over a wide range of *V*_*m*_ (Fig 2A; Table 2). Hence, the SS-approximation for activation *b* computing *I*_*CaT*_ (with normalised conductance) and the full dynamic model is an excellent fit (Fig 4B). However, below resting *V*_*m*_ at around -55 mV, SS approximated currents deviate significantly for the inactivation variable *g* either in solo or combined with the activation variable *b*. Given *τ*_*g*_ is well over 100 ms and rises even higher at hyperpolarised *V*_*m*_, this is unsurprising (Fig 2). Rather unexpected, however, is the agreement for full simulations of the FTM with all these combinations of SS and ODE variables (Fig 4B, lower pane) where Δ*V*_*m*_ is at most about 0.1. Extending solution scans over *V*_*m*_ of the inactivation variable out to *T* = 100 ms sharply improves the fit for *I*_*CaT*_ at or above resting *V*_*m*_ with all SS approximation combinations (upper pane inset, Fig 4B). Even the combined *b* and *g* SS approximation—with no ODEs dynamically simulating the T-type behaviour—we obtain excellent reproduction of *I*_*CaT*_ from resting *V*_*m*_ and up with Δ*I*_*CaT*_ falling dramatically from *O*(0.1) for *T* = 10 ms to *O*(0.01) with *T* = 100 ms. Permitting an adaptive time-stepper to increase *dt* to 0.1 seconds during full FTM solutions, we thus obtain the overall good representation of *V*_*m*_ for the SSP as shown.

#### The L-Type Ca^2+^ channel

**Fig 5:**
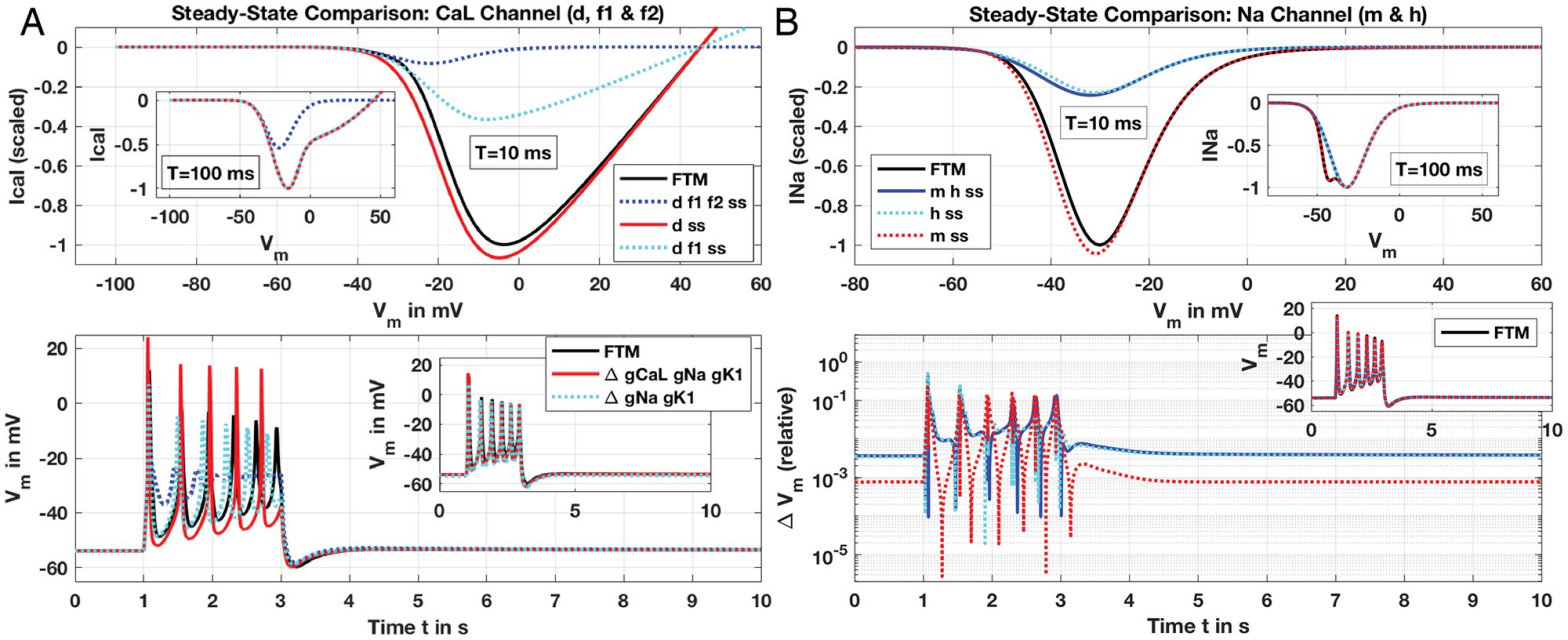
Steady-state approximation comparisons with ODE solutions for *I* − *V* curves and *V*_*m*_ results from FTM simulations; L-type (Panel A) and Na^+^ (Panel B) channels. Full ODE solutions displayed with black traces (labeled ‘FTM’) and hybrid SS-ODE or full SS solutions with dotted/coloured traces as noted. Presented plots similar as for *BK* and T-type, with upper panes displaying currents (scaled to maximum inbound) solved out to *T* = 10 or 100 ms (inset), and lower panes *V*_*m*_ or Δ*V*_*m*_ as labeled. (Panel A) *CaL* channel, *d* activation and *f*_1_, *f*_2_ inactivation variables. *I*_*CaL*_ shown (upper pane) for full ODE (black trace, FTM) and SS approximated variables as noted (blue-, red- and cyan-dotted). SS approximations for only *f*_2_ not performed. *V*_*m*_ presented (lower pane) and not Δ*V*_*m*_ due to obvious deviations; note same trace legend as upper pane (black FTM and coloured SS combinations). SSP simulations with varied conductances (inset) for two SS approximations and improved *V*_*m*_ fit to FTM: *d* and *f*_1_ both SS (cyan trace) with *g*_*CaL*_ reduced; only *d* approximated with SS (red trace) with *g*_*CaL*_ reduced as well as *g*_*Na*_ and *g*_*K*1_ both increased (see text for details). (Panel B). Na^+^ channel, *m* and *h* activation/inactivation variables. Upper pane presents currents for full ODE, hybrid SS and full SS solutions out to *T* = 10 and 100 ms (inset), and lower pane result for SSP showing Δ*V*_*m*_ (relative) and *V*_*m*_ (inset) with same ODE and SS combinations as noted in upper legend: full ODE (black trace), both *m* and *h* activation/inactivation variables SS (blue dotted) and *m* or *h* SS (red and cyan dotted, respectively). See SSP description for numerical details.

Essential to both uSMC function and hence any uSMC model, the L-type Ca^2+^ channel submodel has three variables, one activation (*d*) and two inactivation (*f*_1_, *f*_2_). Time constants for activation *d* and one inactivation *f*_1_ are at or below 10 ms; *f*_2_ meanwhile remains well above to nearly 100 ms over a wide range of *V*_*m*_ (Fig 2A). Currents, *I*_*CaL*_, are produced with the expressions

$$\begin{array}{cc} I_{CaL} & ={\bar{g}}_{CaL}d^{2}f_{Ca}(0.8f_{1}+0.2f_{2})(V_{m}-E_{Ca})\\ d^{ss} & =1/(1+\text{exp}(\frac{-(V_{m}+22)}{7}))\\ f^{ss} & =1/(1+\text{exp}(\frac{V_{m}+38}{7}))\\ \tau_{d} & =2.29+5.7/(1+(\frac{V_{m}+29.97}{9}{)}^{2}),\text{in}\;\text{ms}\\ \tau_{f2} & =90.97(1-1/[(1+\text{exp}(\frac{V_{m}+13.96}{45.38}))(1+\text{exp}(\frac{-(V_{m}+9.5)}{3.39}))]),\text{in}\;\text{ms} \end{array}$$ (8)

with *τ*_*f*1_ fixed at 12 ms, and *f*_*Ca*_ a Ca^2+^-modulating function desensitising the L-type by shifting *V*_*m*_-dependent activation to higher *V*_*m*_ with increasing [Ca^2+^]_*i*_ (see Tong, et al. for full formulations). Both inactivation variables relax to the same steady-state function *f*_*ss*_ at their respective given proportions. *f*_2_ with its slow *τ*_*f*2_ around resting *V*_*m*_ should give a poor SS representation and we thus only tested *f*_2_ approximated by *f*_*ss*_ in combination with the other two variables—that indeed performs inadequately at *T* = 10 ms (see Fig 5A). Only the activation variable, *d*, at the shorter time-span *T* = 10 ms displays reasonable SS approximation that further improves with *T* extended to 100 ms. However, the *f*_2_ SS comparison retains unacceptable deviations from the ODE-solved *I*_*CaL*_ even at this longer timescale, and thus informs the FTM ODE-SS variants tested next.

Modest variations in conductances for the better performing combinations (*d* alone or combined with *f*_1_) improved the reproduction of the full FTM result (Fig 5A lower pane, inset), but required introduction of non-zero Na^+^ conductance. The CellML implementation reproducing Tong, et al.’s Fig 12 runs with an inactivated Na^+^ channel (see Table 1); substantial variations to *g*_*CaL*_ and *g*_*K*1_ stubbornly fail at improving the SS approximations as shown. Alternatively, we do obtain considerable improvements in the qualitative fits with modest variations to conductances in two variant combinations of SS variables: (*i*) activation *d* alone with *g*_*Na*_ = 0.0703, *g*_*K*1_ = 0.535 and *g*_*CaL*_ = 0.39 nS/pF; and (*ii*) *d* and inactivation *f*_1_ with *g*_*Na*_ = 0.0703, *g*_*K*1_ = 0.60 nS/pF and *g*_*CaL*_ at the baseline CellML value of 0.6 nS/pF. All these varied conductances still fall well within physiological ranges as discussed by Tong, et al.

#### The voltage-gated Na^+^ channel

The conductance of the voltage-gated Na^+^*g*_*Na*_ is at most around 1/6 of *g*_*CaL*_ yet shapes the model response to stimulus current, similar to the T-type channel. Raising *g*_*Na*_ from zero (set in the CellML implementation) to the range observed by Tong, et al. increases the number of APs then damps *V*_*m*_ response to a depolarised level with successively larger increases of *g*_*Na*_ up to 0.1 nS/pF. We thus include the Na^+^ channel in our RTM, and given relaxation times of the activation variable *m* of at most 7.2 ms (during depolarisation), a SS approximation for its action looks promising. Current computed for Na^+^ channel includes *m* and the inactivation variable *h*:

$$\begin{array}{cc} I_{Na} & ={\bar{g}}_{Na}m^{3}h\left(V_{m}-E_{Na}\right)\\ m^{ss} & =1/(1+\text{exp}(\frac{-(V_{m}+35.96)}{9.24}))\\ h^{ss} & =1/(1+\text{exp}(\frac{V_{m}+57}{8}))\\ \tau_{m} & =0.25+7/(1+\text{exp}(\frac{V_{m}+38}{10}))\text{in}\;\text{ms}\\ \tau_{h} & =0.9+1002.85/(1+(\frac{V_{m}+47.5}{1.5}{)}^{2})\text{in}\;\text{ms}, \end{array}$$ (9)

where *τ*_*h*_ sharply peaks to nearly 1,000 ms around resting *V*_*m*_, and diminishes prospects for a successful SS approximation of *h*. Current solutions comparing the full ODE and SS evaluations with either both variables or each in solo reflect these relaxation time scales, with *m* SS closely mimicking the full ODE *I*_*Na*_, but not *h* for *T* out to 10 ms (Fig 5B, upper pane). Extending *T* to 100 ms improves all approximations, yet still leaves a deviation around resting *V*_*m*_ for either combination with *h* – as expected given the significant rise for *τ*_*h*_ at that *V*_*m*_ value.

Regardless, deploying these SS evaluations in substitute of the full ODE *I*_*Na*_ calculation for SSP comparisons with the FTM shows excellent qualitative agreement with all combinations of *m* and *h* (Fig 5B, lower pane inset). Relative deviations from *V*_*m*_, or Δ*V*_*m*_, is more revealing, however, of errors rising to order 0.1 for all variations of SS-ODE *m* and *h*. Closer examination of the SSP result shows peaks varying somewhat for each SS-ODE combination, with *m*-SS alone slightly temporally advanced and *h*-SS combinations slightly delayed from the full ODE FTM simulated APs. These slight time-shifts are enough to result in the rather high Δ*V*_*m*_ observed. Given the qualitative agreement using both SS *m* and *h*, we thus include *h* as a SS approximation in the RTM.

Thus far, of the four channel submodels considered here for SS approximation, the *BK*, T-type, L-type and *V*_*m*_-gated Na^+^, all include nine activation and inactivation variables. Of these variables, only the L-type inactivation *f*_2_ appears unsuitable for SS approximations with its relaxation time reaching up to about 100 ms and the inactivation *h* for the Na^+^ channel peaking to nearly 1 second (Fig 2). Preliminary results support exclusion of *f*_2_ but inclusion of *h* for SS substitutions, however, likely due to the peak of *τ*_*h*_ confined to *V*_*m*_ around resting (Fig 5). Alternatively, notice *τ*_*f*2_ remains well above 10 ms throughout the AP event and hence performs poorly with a SS representation. We thus apply a SS approximation to eight of the dynamic variables and eliminate in turn eight ODEs from the FTM in our RTM. This results in a reduction from 19 ODEs in the FTM into 5 for the RTM thanks to six variables removed using *I*_*K*1_ as representative of three other K^+^ channels and SS approximations eliminating eight. Twelve less dynamic variables in the system should hold significant impact on the computational performance of the RTM which we consider next.

## Results

### RTM performance in reproducing experiments

Individually, each modification to the FTM presented so far is promising, requiring either no or modest alterations to the model parameters listed in Table 1 as provided with the CellML implementation. It is the collective behaviour that is of most import, however, and here we present each modification explored above into a final assembled RTM aimed at reproducing the FTM behaviour but simplified and with reduced computational expense.

#### The SSP

**Fig 6:**
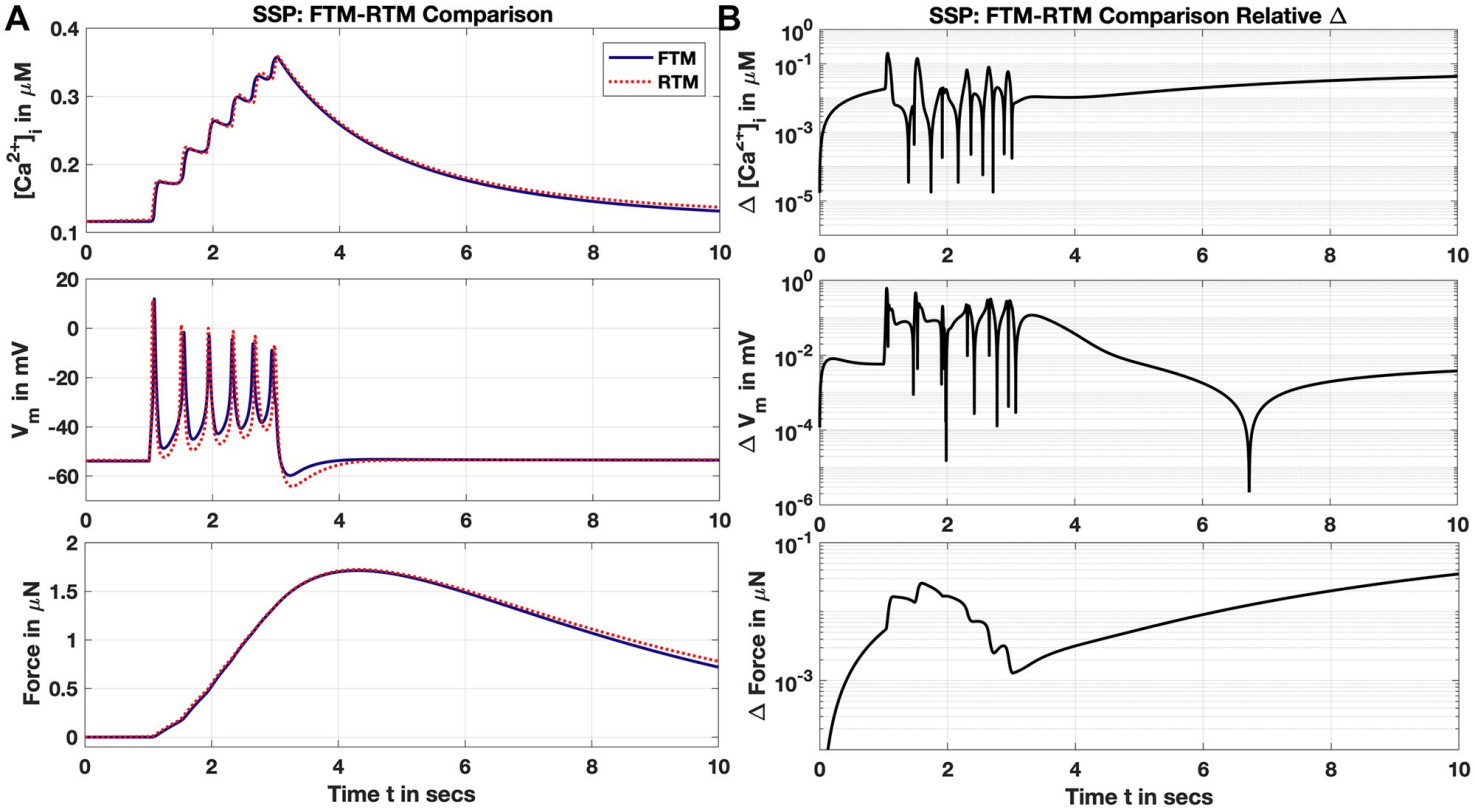
SSP RTM reproduction and comparison with FTM. (Panel A) Complete RTM (red dotted) with all modifications detailed in text presented in comparison with FTM (blue trace), showing [Ca^2+^]_*i*_ (upper pane), *V*_*m*_ (middle pane) and computed Force (lower pane) as dependent on [Ca^2+^]_*i*_ (see text). (Panel B) Relative difference for results presented in Panel A. Note semilog scales for all. Key RTM parameters include: *g*_*CaL*_ = 0.6, *g*_*Na*_ = 0.0895, *g*_*K*1_ = 0.7 pA/pF and *β* = 0.0169.

Initially, we show effectiveness of the RTM in reproducing the SSP so far utilised as a test comparison with the full FTM, and present *V*_*m*_, [Ca^2+^]_*i*_ and force generation for the RTM (Fig 6). The FTM calculates force as dynamically dependent on [Ca^2+^]_*i*_ with a familiar ODE tracking deviation from a steady-state:

$$\begin{array}{cc} \frac{d\omega }{dt} & =\frac{\omega^{ss}-\omega }{\tau_{\omega }}\\ \omega^{ss} & =1/(1+\frac{K_{m,F}}{{\left[Ca^{2+}\right]}_{i}}{)}^{nF}\\ \tau_{\omega } & =4000(0.235+\frac{1-0.235}{1+({\left[Ca^{2+}\right]}_{i}/K_{m,F}{)}^{nF}})\left(\text{in}\;\text{ms}\right), \end{array}$$ (10)

with half-maximal activation at around 160 nM [Ca^2+^]_*i*_, and Hill coefficient about 3.6 (*via* the CellML implementation). Unfortunately, the time scale, *τ*_*ω*_, is quite long at 4 seconds and higher as [Ca^2+^]_*i*_ rises during stimulus, precluding application of a SS approximation for *ω* here.

Nevertheless, we obtain qualitatively excellent reproduction of the FTM with our RTM for this SSP instance (Fig 6A), with reasonable relative deviations throughout (Panel B). The RTM again excludes three K^+^ channels (Sec. Elimination of Channels Based on Conductance) as well as the *N*/*K* exchanger, and eight ODEs using SS approximations (Sec. Steady-state Approximation) including the inactivation *h* for the Na^+^ channel. Modest alterations to the conductances listed in Table 1 as noted in Fig 6 produced this result. Although *V*_*m*_ peaks and valleys of the RTM moderately exceed or overshoot the FTM during stimulus and recovery, resulting impact on [Ca^2+^]_*i*_ where the RTM falls below the FTM is mitigated with a straightforward alteration to *β*. Raising the free-buffer fraction of [Ca^2+^]_*i*_ up from 0.015 to 0.0169 enabled the tight correspondence for the [Ca^2+^]_*i*_ presented, and provides the superb result for force generation as computed from [Ca^2+^]_*i*_. This raised *β* moreover still remains lower than that used by the Tong, et al. cited model of Standen & Stanfield with their *β* set to 0.05 for neuronal axons [17].

#### Plateaus, bursting and sustained APs

**Fig 7:**
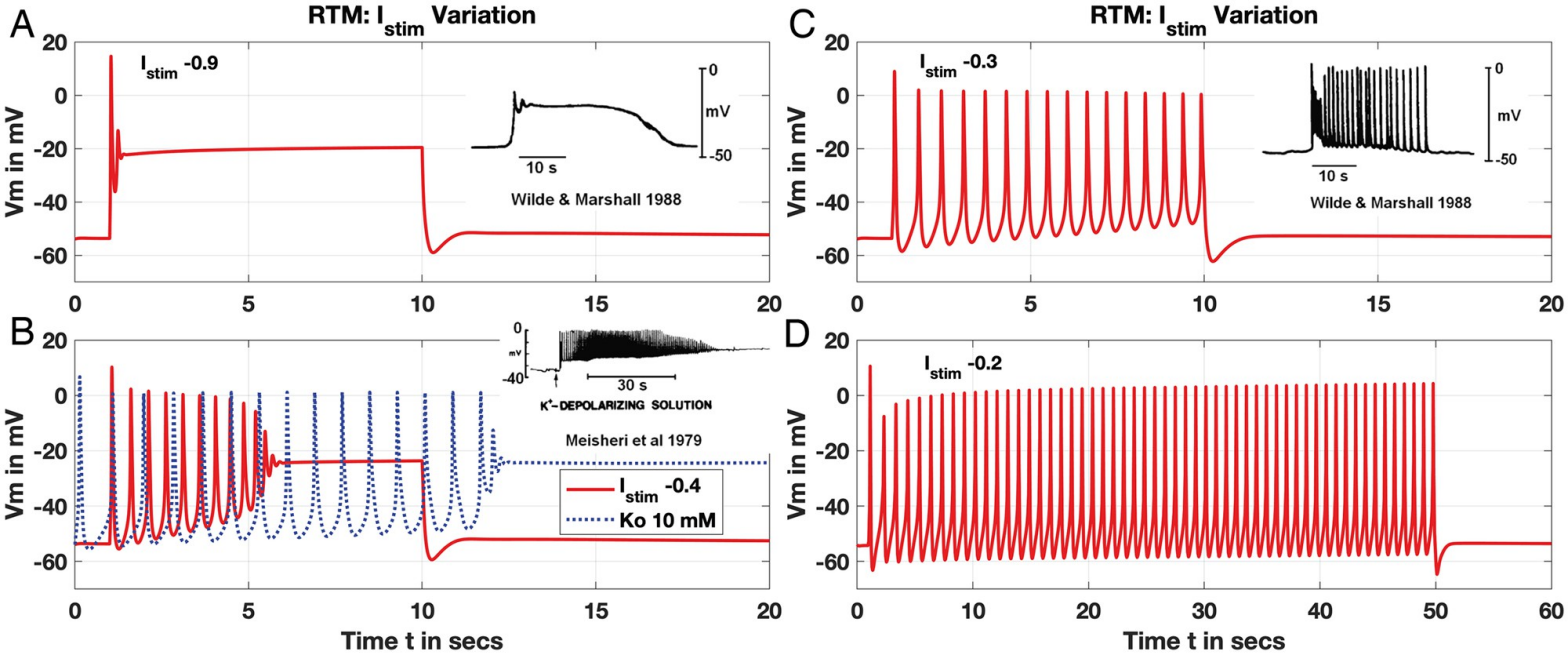
RTM: Variation of *I*_*app*_. Comparison results from Tong, 2011 Fig 11A, Fig 11B and Fig 11C and Tong, 2014 Fig 8D. Stimulus strength varied as noted aimed at reproducing experimental results (insets, Panels A-C) as given in Tong, 2011 Fig 11, or extended bursting (Panel D). Each *I*_*app*_ applied up to *t* = 10 s starting at *t* = 1 s, except Panel D out to *t* = 50 s. Additional simulation with elevated extracellular initial condition K^+^ at 10 mM for comparison and no applied stimulus (blue dotted, Panel B). Conductances for key currents: *g*_*CaL*_ = 0.6, *g*_*Na*_ = 0.0895, *g*_*K*1_ = 0.7 pA/pF (identical to SSP values, Panels A-C), with *g*_*K*1_ = 0.75 for Panel D. Experimental traces (insets) adapted from Tong, et al. 2011 Fig 11.

The promising fit to the SSP with the RTM may be due to the SSP utilised as a test case for each modification considered in turn above. We thus exposed the RTM to additional scenarios, specifically, Tong et al.’s Fig 11 of 2011 [12] and Fig 8 of Tong, et al.’s 2014 [16]. Varying *I*_*app*_ from a strong down to a mild current produces the array of AP shapes and responses shown in Fig 7. For high-enough *I*_*app*_, a plateau-type AP forms with *V*_*m*_ settling into an elevated steady state comparable to observation of Wilde & Marshall [25] (Fig 7A). Lowering *I*_*app*_ then generates a burst of APs that plateau at depolarised *V*_*m*_ analogous to Meisheri, et al. [26], that also emerges with a depolarising ‘pulse’ of initially elevated extracellular K^+^ and no applied stimulus (Fig 7B). Further drops of stimulus strength demonstrates sustained APs for the duration of *I*_*app*_ either over 10 or 50 secs (Fig 7C and 7D), analogous to Wilde & Marshalls report [25]. Each conductance and parameter of the RTM is moreover identical to those utilised for the SSP comparison result except for the depolarising K^+^ pulse. Only one other exception occurs, however, with the longer-duration *I*_*app*_ over 50 s requiring a higher *g*_*K*1_ of 0.75 pA/pF.

Over a longer duration *I*_*app*_, the 2011 FTM failed to generate the AP bursting profile shown here produced by the RTM in Fig 7D. This complication with the 2011 model version inspired later additions of the four K^+^ channels noted as such in Fig 1 by Tong, et al. with their 2014 model [16]. Increasing the *g*_*K*1_ as described by Tong, et al. from the CellML value of 0.52 to 0.64 pA/pF improved the FTM response, elongating an otherwise short burst of APs before damping into the plateau phase (see Fig 2 of [16]). However, the FTM required rescaling the already quite slow *K*1 time constants by substantial factors, i.e., 2 × *τ*_*q*_, 20 × *τ*_*r*1_ and 20 × *τ*_*r*2_—pushing them as high as 5e6 ms (see Table 2)—in order to generate the longer AP burst we obtain here by simply increasing *g*_*K*1_ to 0.75 pA/pF in our RTM.

### Computational performance

For solutions of the FTM in SSP conditions and each variant of reduction detailed in methods—substituting one K^+^ channel for three others and SS approximations for ion channel submodels—and the full RTM encompassing all these reductions, we present performance details in Table 3. Over a sample of *n* = 10 runs, the average time-to-solve per variant improves modestly for the representative *K*_1_ variant, and increases for each channel submodel with SS approximations all to around the same improvement factor of 1.7. Combined together into the final assembled RTM, we observe that the RTM is, on average, 2.1 times faster to solve for a single cell than the FTM. The individual factors of improvement are not cumulative. This is likely due to internal mechanisms beyond user control with the CVODE solver.

**Table 3:** Comparison time-to-solve the SSP. FTM performance and each variant of reduction including conductance replacement (*K*_1_ representative of *K*2, *Ka* and *h*), individual ion channel submodel SS approximations as noted, and the full RTM with all reduced items presented. Solutions of the single cell SSP performed in OpenCOR using CVODE with maximum *dt* = 0.1, tolerances (relative/absolute) at 1*e*^−7^, with *n* = 10 trials each on a macbook pro laptop. Solutions of 2D and 3D tissue SSP trials performed using CHASTE with CVODE (*dt*_*max*_ = 0.1 with default settings) on a single-thread of Intel Xeon 6254 CPU with *n* = 10 trials. Average time to solve (*μ*) and standard deviation for the set of trials (*σ*) provided as noted. Percent improvement calculated as change relative to results with FTM, i.e., abs(*t*_*FTM*_ − *t*_*RTM*_)/*t*_*FTM*_.

Model/Submodel	Time to solve (ms, *μ*, *σ*)	% Improved (*μ*, *σ*)	Factor Improved (*μ*)
FTM	328.5; 37.0	n/a	n/a
*K*1 Representative	253.5; 28.8	22.8; 2.6	1.3
*BK*	192.8; 8.9	41.3; 5.4	1.7
T-type	196.9; 19.3	40.1; 1.7	1.7
L-type	190.0; 2.9	41.9; 7.3	1.7
Na^+^	203.6; 9.2	38.0; 5.3	1.6
RTM	154.2; 1.1	53.1; 6.5	2.1
FTM (2D)	71.5; 0.65	n/a	n/a
RTM (2D)	27.3; 0.32	62; 0.1	2.6
FTM (3D)	914.5; 10.4	n/a	n/a
RTM (3D)	248.8; 9.9	73; 1.2	3.7

Pilot simulations on both 2D and 3D geometries comparing the FTM and RTM in a monodomain tissue model were conducted to assess scalability—as described in Methods. The RTM again proves faster with factor improvements of 2.6 and 3.7 speedups in the 2D and 3D geometries, respectively (see Table 3), using the default CVODE options in CHASTE [21]. This follows from the reduced suite of ODEs in the RTM. For the 2D simulation, a total of 121 nodes × 5 ODEs gives 605 unknowns per time step versus 121 × 19 or 2299 per time step in the FTM. Similarly for the 3D simulation, the RTM solved 9550 unknowns versus the FTM’s 36,290 per time step.

## Discussion

We presented our steady-state approximation method for reducing the order of a uSMC model by considering the multiple time scales inherent in a published model by Tong, et al., 2011 [12]. Our final assembled RTM demonstrates both accurate reproduction of experimental results while substantially improving computational performance. Arriving at this RTM does require careful adjustments of parameters such as conductance strengths or buffering fractions and numerical experimentation. Nevertheless, it is a straightforward process, particularly compared with formal sensitivity and bifurcation analysis [8]. Evaluating overall time-scales for each submodel within the context of the system of interest is essential. Importantly, in the uSMC model considered here, the activity of the physiological system is over multiple seconds and longer while individual channel dynamics may settle to steady-state at orders of magnitude faster [9].

Observing multiple time scales in a model system and exploiting steady-states for relatively fast sub-mechanisms is not unknown and indeed formed the basis for many systems including the widely-used FN neuronal AP model [5]. The seminal ventricular cardiac model of Luo and Rudy, for instance, incorporated a steady-state activation function for their time-independent K^+^ channel [27, 28]. With a *τ* of 0.7 ms, it operates considerably faster than other channels in their model with inactivations relaxing up to 100 ms. Moreover, the Luo & Rudy sequence of papers simulate cardiac behaviour over hundreds of milliseconds which is quite suitable for a cell model expressing ion channel time scales of 10 to 100 ms. Subsequent multi-scale models of the heart built on the Luo & Rudy cell model investigate similar temporal ranges [29], as do other multi-scale efforts founded on different cell models. Recent organ models such as from Margara, et al. [1] deploy the Tomek, et al. ventricular myocyte model [15] itself based on the Grandi, et al. model [30] that includes an ODE with a *τ* for a Na^+^ channel relaxing within 0.17 ms. Resolving fully three orders of magnitude faster than the simulated scales of the organ model, such an ion channel mechanism is a ripe candidate for steady-state approximation.

Cardiac electrophysiology models using bidoman/monodomain methods [31] laid the groundwork for their application to other organs, such as the gastrointestinal system [2]. Coupling partial differential equations for electrical diffusion with systems of intracellular ion dynamics, these models are computationally demanding; any simplifications are eagerly sought and cell-level models are a natural target. For instance, the Corrias & Buist model of the gastric pacemaker, the interstitial cells of Cajal (ICC), incorporates relaxation times spanning from just under 10 ms (for Na^+^and K^+^ channels) up to nearly 100 ms (L-type Ca^2+^) [14]. Du. et al. simplified the Corrias & Buist model in their later organ-scale model of gastric electrophysiology, investigating slow-waves of activity over scales of seconds up to minutes [32]. Yet, the ICC Na^+^ channel activation variable with a *τ* of 10 ms remained in the full multi-scale model [2]. Approximating such rapid millisecond-scaled dynamic mechanisms with their steady-state evaluations in a seconds-to-minutes simulation is easily overlooked. As demonstrated here, such approximations—for suitable time scales of interest—hold considerable potential for improving computational performance.

Sensitivity & bifurcation analysis sifting out model components while maintaining essential dynamics as applied to the uSMC model of Rihana, et al. [11] are certainly important and valid approaches. Reduced from the original ten dynamical variables, Laforet, et al. utilised the reduction of Parthimos et al.’s arterial model [8] and the Rihana, et al. reduction [33] to produce a three-variable uSMC cell model [34] slated for later use in that group’s full 3D multi-scale model of the uterus [3]. The relative simplicity of their minimal uSMC cell model enabled greater flexibility to explore otherwise computationally infeasible simulations—but only after substantial exertion for the cell model analysis. Of note, we considered performing a steady-state reduction of order with the Rihana, et al. model, but neither that nor subsequent versions published parameter values for the relaxation *τ*’s [11].

With the Tong, et al. 2011 model, after removing several K^+^ channels, using the *K*_1_ as a representative K^+^ channel, and the steady-state approximations shown, we eliminated eight dynamical variables out of the total thirteen in our RTM. The relative conductance strengths listed in Table 1 informed selection of candidates for elimination starting with the second-strongest conductance channel, *K*_*a*_, and progressing through the rest. Interestingly, despite ${\bar{g}}_{Ka}$ nearly an order of magnitude stronger conductance than ${\bar{g}}_{K2}$, *K*_2_ emerged as the more relevant channel (compare Fig 3B with Fig 3A). *K*_2_ relaxes over the course of seconds while the *K*_*a*_ does within about 50 ms (compare Fig 6E and 7C, [12]). Hence, both conductances and time scales dictate suitability for this representative channel approach.

Observing the relative conductance strengths in any suitable system—with an eye to relative dynamic behaviour—permits reproduction of this representative channel approach in the following manner: (*i*) Enumerate conductances according to strength; (*ii*) Evaluate relative contribution to dynamics; and (*iii*) Adjust dominant or representative conductance. Certainly, this method may be combined with a sensitivity and bifurcation analysis to reduce our RTM even further, with the corresponding effort involved.

Meanwhile, the relative simplicity of comparing time scales for individual sub-model variables with the overall time scale of interest also suggests the possibility of automation. Algorithmically, determining suitability of steady-state approximations within a modelling framework appears a pragmatic possibility, perhaps as a precursor to a full reduction analysis, and may be similar to the following procedure:

- Set time scale of interest, *T*_*i*_, (here in seconds);
- Set membrane voltage range of interest, *V*_*span*_, (here ∼ [−50, 50] mV);
- Identify potential variables for SS approximation:
  - If *τ*_*x*_ ≤ 0.01 × *T*_*i*_ for *V*_*m*_ ∈ *V*_*span*_
  - Test corresponding variable, *X*, for SS performance in channel model:
    - Select time step size as appropriate for comparison; i.e., *dt* = 0.01 ms;
    - Perform full ODE simulation channel model;
    - Compare ODE result with SS variable approximation.

The above procedure simply replicates what we conducted here in our SS explorations, including dynamic variables that performed well with SS approximations despite relaxation rates well exceeding our ‘rule of thumb’ guide for 10 ms. As seen in the plots for the Na^+^ variable *h* and the T-type variable *g* in Fig 2, this is due to the peak *τ* levels sitting at the lower end of *V*_*span*_. Hence, by specifying these temporal and voltage spans of interest, an automated system may identify potential dynamic variables for SS approximations and evaluate their suitability with full ODE and SS comparisons comparable to those conducted here.

### Limitations

Representative channel usage such as the *K*_1_ in our RTM here, does entail some disadvantages, however. Although for our purposes we reproduced the test cases shown for the SSP and variations of *I*_*app*_, there may be scenarios where the RTM will fail without the full bank of K^+^ channels in the FTM. By the same token, there may also be scenarios where the FTM would fail given the extraordinary array of K^+^ channels discovered in the exhaustive transcription maps of Atia, et al., for instance [23]. These possibilities are simply unknown, particularly given the uncertainties and rather interesting questions as to why so many ion channels with their subtle or dramatic differences are expressed in uSMC, or any cell type for that matter.

### Modeling implications: Realism and relevance

Our reduction reinforces how models replicating measured data do not require every mechanism known to reside in a cellular system. Note the expanded 2014 FTM utilising nine K^+^ channels still omitted numerous examples of the 22 channels reported by Atia et al. [23]. Regardless, the FTM—without this full suite of channels—reproduced the measured data that we also accomplished here with even fewer mechanisms in the RTM. Certainly, excluding channels observed in a cell reduces the molecular fidelity of any mathematical model regardless of the cellular system under study. However, fidelity must necessarily be balanced against tractibility and relevance.

Mathematical models of the biological system are constrained by the available measured data; our ‘minimal model’ presented here reproduces the available data. Of course, as with any model, if in the future the model failed to reproduce new data, molecular mechanisms could be reintroduced systematically to determine which drive the particular novel behaviour. This is a particular benefit of a reduced modeling strategy, in that it provides important insights into the required mechanisms for different cell behaviours, teasing out their relative influences.

An example of a mechanism that may be important for some behaviours and not others is the ryanodine receptor Ca^2+^ channel (RyR). It is clearly expressed in the uSMC, but also appears to be functionally inert for the behaviours observed [35, 36]. Reports of the RyR’s appearance in the uSMC likely inspired its incorporation in a Ca^2+^ dynamical uSMC model that also excluded the other intracellular Ca^2+^ channel, the inositol trisphosphate receptor (IP3R) [37]. Unlike the RyR, the IP3R not only appears in the uSMC, it does appear influential on uterine contractile activity [9]. Striking a careful balance between accuracy and tractability, ‘minimal models’ aid illuminating the relevance of resident cellular mechanisms but of course are always constrained by the experimental scenarios providing the data.

### Time scales and performance

Suitability of the steady-state approximation approach is of course dependent on the time scales of interest, or *T*_*i*_. For overall behaviour of an organ-scale model with relatively similar time scales on the ion channel level as, say, with the Luo & Rudy cardiac model, a steady-state substitution may fail to perform. Alternatively, with longer organ-scale phenomena as with the uterus over multiple seconds and more, the extraordinarily rapid ion channel relaxation times as in the FTM prove excellent candidates. Careful consideration of the relative time scales and particularly the context for the overall system of interest naturally dictates whether elimination of dynamic variables will succeed—as described above.

If indeed the relative time scales prove suitable as demonstrated here with the RTM, steady-state replacements of full dynamical variables combined with modest calibrations to parameters in the model readily provide substantial improvements in computational speed while maintaining accuracy. For a single cell model, a factor of two reduction in time to solve may be negligible—150 ms versus 300 (Table 3). However, distributed over significant numbers of grid points in a full organ-scale model this difference is amplified considerably, potentially producing significant reductions in absolute computation times. Hence, we trialed pilot simulations of a monodomain tissue model on simple 2D and 3D geometries (square and hollow cylindrical tube, respectively) with the improved times-to-solve as noted exceeding the single-cell simulation improvements (Table 3). Of course, different implementations, solvers and settings will produce different results. Regardless, reducing the number of unknowns per time step by roughly a factor of 4, as here with the RTM, will reduce computational load and enable greater flexibility in parameter sensitivity analyses and *in silico* investigations.

## Conclusion

A reduction of order was performed for the Tong, et al. uSMC model utilising steady-state approximations for rapidly resolving dynamical variables. The relatively slow functions of uSMCs over the scale of seconds and longer provide the key context for eliminating whole ODEs with millisecond-level time scales. Examining these relative time scales and testing suitability for reproducing observed uSMC yields the RTM presented here. Demonstrating the effectiveness and relative ease of a steady-state reduction of order approach with this uSMC model test case underlines its utility for the numerous cell models deployed in myriad multi-scale representations, and holds great potential for expanding the computational possibilities for simulating the many intrinsically multi-scaled organs and systems.
